# Supplementary material for: Treponema pallidum among Female Sex Workers: A Cross-Sectional Study Conducted in Three Major Cities in Northern Brazil
Source: Pathogens. 2021 Jul 22;10(8):923. doi: 10.3390/pathogens10080923 (PMC8401545; doi:10.3390/pathogens10080923)
Supplement: Supplementary file 1 [file pathogens-10-00923-s001.zip › pathogens-1250270-supplementary.pdf]

SUPPLEMENTARY MATERIAL

Title: *Treponema pallidum* among female sex workers: a cross-sectional study conducted in major cities in northern Brazil.

Authors: Luiz Fernando Almeida Machado, Jacqueline Cortinhas Monteiro, Leonardo Quintão Siravenha, Marcelo Pereira Mota, Marlinda de Carvalho Souza, Adalto Sampaio dos Santos, Márcio Ronaldo Chagas Moreira, Rogério Valois Laurentino, Aldemir Branco Oliveira-Filho, Maria Alice Freitas Queiroz, Sandra Souza Lima, Ricardo Ishak, Marluísa de Oliveira Guimarães Ishak.

Table S1. Factors not associated with exposure to *Treponema pallidum* among female sex workers in northern Brazil using bivariate and multivariate analyzes.

Factors	N (Total sample)	N (Exposure +)	Bivariate OR (95% CI)	Multivariate OR* (95% CI)
Over 35 years versus (vs.) Up to 35 years	173	59	0.9 (0.5–1.3)	1.1 (0.6–1.7)
Not married (Single + Divorced/Widowed) *vs.* Married	345	124	1.1 (0.6–1.5)	1.5 (0.8–2.0)
Sexual partner from another Brazilian state *vs.* Local partner only	249	91	1.1 (0.7–1.5)	1.3 (0.8–2.1)
Sexual partner from another country *vs.* Local partner only	146	51	0.9 (0.5–1.4)	1.1 (0.7–1.9)

†Last 12 months. OR: Odds Ratio. 95% CI: 95% confidence interval. OR: Odds Ratio. *OR not adjusted. The average age and the average number of partners per week were used to form FSW groups in these analyzes.
